# The complete chloroplast genome sequence of *Crassocephalum crepidioides* (Benth.) S. Moore. (Asteraceae)

**DOI:** 10.1080/23802359.2021.1934165

**Published:** 2021-06-03

**Authors:** Song Wei-Cai, Liu Xiao-Fan, Shuo Wang, Zhang Rui-Qing, Shi Chao, Yun-Jiao Zhang

**Affiliations:** a College of Marine Science and Biological Engineering, Qingdao University of Science and Technology, Qingdao, China; b Kunming Medical University Haiyuan College, Kunming, China

**Keywords:** Asteraceae, *Crassocephalum crepidioides*, chloroplast genome, phylogenetic relationship

## Abstract

The complete chloroplast (cp) genome of *Crassocephalum crepidioides* was sequenced and assembled for the first time. In this study, the total genome size is 150,596 bp in length and demonstrates a typical quadripartite structure containing a large single copy (LSC, 82,575 bp) and a small single copy (SSC, 18,293 bp), separated by a pair of inverted repeats (IRa, IRb) of 24,864 bp. The G + C content of this cp genome was 37.21%. Gene annotation analysis identified 130 genes including 85 protein-coding genes, 37 transfer RNA, and 8 ribosomal RNA genes. The maximum-likelihood phylogenetic analysis result showed that *C. crepidioides* was closely related to *Nannoglottis ravida* in the phylogenetic relationship.

Chloroplasts is a double membrane-bounded organelle in of plants. It plays important metabolic roles, including photosynthesis, amino acid and lipid synthesis (Daniell et al. 2016; Mehmood et al. 2020a). Chloroplast genomes are inherited from the mother, which helps to study phylogenetic relationships (Mehmood et al. 2020b). *Crassocephalum crepidioides* (Benth.) S. Moore. (Henderson 1973) is an annual edible plant that is widely distributed in tropical and subtropical areas (Rajesh 2011). It is an erect, less branched herb, about 40–100 cm tall. The stem is stout, soft, angular, apex with short, thick hair (Kostermans et al. 1987), the leaves are elliptic to ovate, and the seeds consist of floating balls of many silky white hairs, which can be blown away by the wind. This plant grows abundantly in tree crop plantations (Dairo and Adanlawo 2007). Edible leaves and stems are often used to treat indigestion or as a laxative (Asif 2016). Also, extracts from this plant have been shown to have chemoprophylactic and anti-inflammatory properties for cancer (Hou et al. 2007). Studying the chloroplasts of *C. crepidioides* is of great significance to further taxonomic and population genetics studies of the species (Ahmed et al. 2012; Guo et al. 2017).

Fresh leaves of *C. crepidioides* were collected from Panlong District, Kunming City, Yunnan Province, China (24°23'N, 102°10'E), and the voucher specimen and DNA were deposited at Qingdao University of Science and Technology (Chao Shi, chch1111@aliyun.com) under a voucher number: HY0516. Total genomic DNA was extracted from fresh leaves using modified CTAB (Allen et al. 2006), the high-quality DNA was sent to construct a genomic library and sequenced using the Illumina HiSeq platform in Novogene (Nanjing, China). About 4.5 Gb high quality, 2 × 150 bp pair-end reads were obtained and were used to assemble the complete chloroplast genome of *C. crepidioides* (Wang et al. 2018). The rbcl gene of *C. crepidioides* (Genbank accession no. MN268502) was used as a seed to assemble the complete chloroplast genome of *C. crepidioides* (Genbank accession no. MW362305) by NOVOPlasty4.2 (Dierckxsens et al. 2017). We also deposited the raw sequencing reads in SRA with Accession no. SRR13823287. Gene annotation was performed with the GeSeq (Michael et al. 2017) and manually corrected for codons and gene boundaries using the Sequin.

The complete chloroplast genome reported here is 150,596 bp in length and exhibits a typical quadripartite structure, consisting of a pair of inverted repeat regions (IRa and IRb) with same length (24,864 bp) separated by the large single copy (LSC, 82,575 bp) and small single copy (SSC, 18,293 bp) regions. The overall GC content is 37.21%, and the corresponding values of the LSC, SSC and IR regions are 35.35%, 30.31%, and 42.84%, respectively. The chloroplast genome of *C. crepidioides* comprised 130 genes, including 85 protein-coding genes, 37 transfer RNA, and 8 ribosome RNA. Noticeably, nine protein-coding genes (rps16, rpoC1, atpF, petB, petD, rpl16, rpl2, ndhB, and ndhA) were disrupted by one intron, and three genes (clpP, rps12, and ycf3) by two.

An alignment comprising the complete chloroplast genome sequences of *C. crepidioides* and other 17 related taxa of Asteraceae was performed in MAFFT version 7.407 (Nakamura et al. 2018; Yupeng et al. 2020). *Nymphoides crenata* was selected as the outgroup of the phylogenetic tree (Journal 2011). Model selected process in Mega version X (Kumar et al. 2018), and GTR + G + I was selected as the optimal model by the Akaike Information Criterion. Phylogenetic tree was constructed using the maximum-likelihood (ML) method and the bootstrap was set to 1000 times iteration in the Mega version X (Figure 1). The phylogenetic analysis (ML) results clearly showed that *C. crepidioides* was belonged to Asteraceae and closer to *Nannoglottis ravida* and *Heteroplexis incana*, these findings further enriched the phylogenetic relationship of the family Asteraceae and will provide useful genetic information for promoting the evolutionary studies of Asteraceae species.

**Figure 1.**
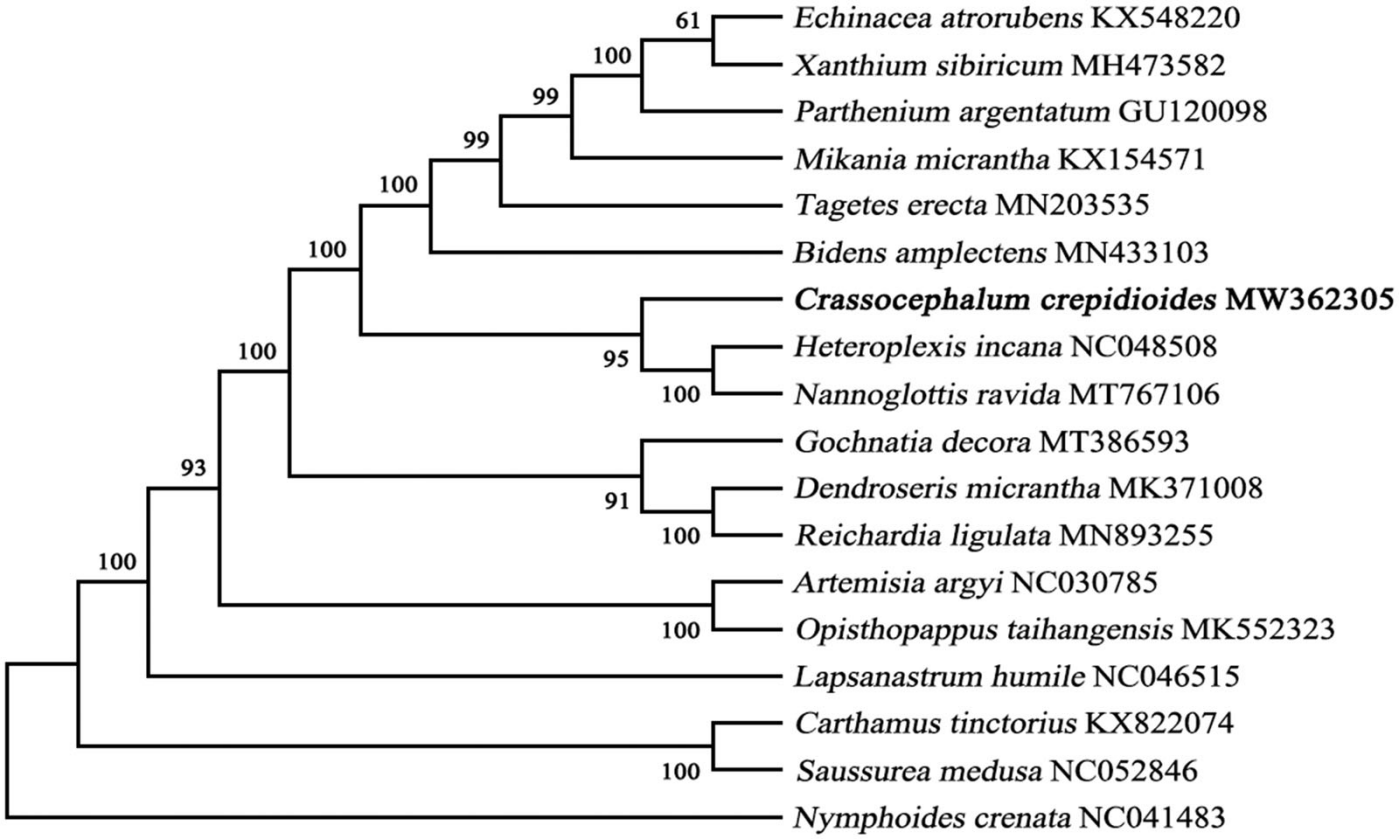
A maximum-likelihood (ML) tree illustrates the phylogenetic position of *C. crepidioides* among part of Asteraceae species. The number on each node indicates bootstrap support value. After species is the chloroplast genome sequence login number used by GenBank.

## Data Availability

The genome sequence data that support the findings of this study are openly available in GenBank of NCBI at (https://www.ncbi.nlm.nih.gov/) under the accession no. MW362305. The associated BioProject, SRA, and Bio-Sample numbers are PRJNA705820, SRR13823287, and SAMN18103693, respectively.
